# Evaluation of the National Notifiable Diseases Surveillance System for Dengue Fever in Taiwan, 2010–2012

**DOI:** 10.1371/journal.pntd.0003639

**Published:** 2015-03-20

**Authors:** Caoimhe McKerr, Yi-Chun Lo, Obaghe Edeghere, Sam Bracebridge

**Affiliations:** 1 Field Epidemiology Training Programme, Public Health England, London, United Kingdom; 2 Field Epidemiology Service, Public Health England, United Kingdom; 3 Office of Preventive Medicine, Centers for Disease Control, Taipei, Taiwan; Australian National University, AUSTRALIA

## Abstract

**Background:**

In Taiwan, around 1,500 cases of dengue fever are reported annually and incidence has been increasing over time. A national web-based Notifiable Diseases Surveillance System (NDSS) has been in operation since 1997 to monitor incidence and trends and support case and outbreak management. We present the findings of an evaluation of the NDSS to ascertain the extent to which dengue fever surveillance objectives are being achieved.

**Methodology:**

We extracted the NDSS data on all laboratory-confirmed dengue fever cases reported during 1 January 2010 to 31 December 2012 to assess and describe key system attributes based on the Centers for Disease Control and Prevention surveillance evaluation guidelines. The system’s structure and processes were delineated and operational staff interviewed using a semi-structured questionnaire. Crude and age-adjusted incidence rates were calculated and key demographic variables were summarised to describe reporting activity. Data completeness and validity were described across several variables.

**Principal Findings:**

Of 5,072 laboratory-confirmed dengue fever cases reported during 2010–2012, 4,740 (93%) were reported during July to December. The system was judged to be simple due to its minimal reporting steps. Data collected on key variables were correctly formatted and usable in > 90% of cases, demonstrating good data completeness and validity. The information collected was considered relevant by users with high acceptability. Adherence to guidelines for 24-hour reporting was 99%. Of 720 cases (14%) recorded as travel-related, 111 (15%) had an onset >14 days after return, highlighting the potential for misclassification. Information on hospitalization was missing for 22% of cases. The calculated PVP was 43%.

**Conclusions/Significance:**

The NDSS for dengue fever surveillance is a robust, well maintained and acceptable system that supports the collection of complete and valid data needed to achieve the surveillance objectives. The simplicity of the system engenders compliance leading to timely and accurate reporting. Completeness of hospitalization information could be further improved to allow assessment of severity of illness. To minimize misclassification, an algorithm to accurately classify travel cases should be established.

## Author Summary

In Taiwan, around 1,500 cases of dengue fever are reported annually. Surveillance and outbreak preparedness are important activities aimed at reducing the burden of dengue fever worldwide. A national web-based Notifiable Diseases Surveillance System (NDSS) for dengue fever has been established since 1997 in Taiwan to monitor trends, and support case and outbreak management. We evaluated this surveillance system based on the Centers for Disease Control and Prevention guidelines and operational staff interviews to ascertain the extent to which stated objectives are being achieved. The results indicate that the NDSS for dengue fever surveillance works well, is easy to use, and can be used to accurately identify cases. The findings have informed recommendations to improve surveillance and its use in outbreak response, including efforts to improve data completeness. Comprehensive surveillance exemplified by the NDSS for dengue fever in Taiwan is necessary for establishing a clear picture of the local burden and distribution of dengue fever to effectively support public health response.

## Introduction

Dengue fever is an acute mosquito-borne viral infection caused by the dengue virus (DENV) that imposes considerable morbidity globally, particularly in the tropics and subtropics [1–3]. DENV is transmitted by several mosquito species within the genus *Aedes*, principally *A*. *aegypti* although outbreaks have been also attributed to the less efficient vector *A*. *albopictus*. In non-endemic settings, imported cases are often reported and the presence of a viable vector in the environment can present a serious threat of autochthonous transmission. Although dengue fever is non-endemic in Taiwan, the vector is abundant and the rising incidence of dengue fever in Southeast Asia in recent years has led to an increase in indigenous cases in Taiwan [4]. Annually, around 1500 cases of dengue fever are reported and there has been an increase in the occurrence of annual epidemics since the 1990s and early 2000s [1]. Transmission occurs as intermittent epidemics, sometimes with intervals of several years, with the main focus of activity in southern Taiwan which is more conducive to dengue outbreaks because of its tropical climate and the presence of *A*. *aegypti* [5].

The Notifiable Diseases Surveillance System (NDSS) has been in operation in Taiwan since 1997 and provides a national web-based platform for reporting and monitoring notifiable diseases, including dengue fever. The objectives of dengue fever surveillance include monitoring trends in incidence and supporting case management, although it may also facilitate outbreak detection. Before 2009, Taiwan Centers for Disease Control (TCDC) used only a clinical case definition for reporting dengue. Many endemic countries have relied heavily on clinical case definitions which may lead to overreporting of cases due to a wide spectrum of clinical manifestations and other circulating viruses [6]. TCDC’s current dengue case definition has been established since 2009, requiring laboratory confirmation for reported cases, which has been shown in other systems to improve specificity [6,7]. Other changes made to the NDSS to optimize the surveillance objectives have included the ability to link complementary datasets, such as laboratory, vector surveillance, and geographic information system data to improve monitoring and early detection of transmission. The integration of multiple surveillance systems has been demonstrated to reduce dengue spread and associated morbidity [7–8].

Dengue fever surveillance through the NDSS was last evaluated by the TCDC in 2009 prior to the introduction of these changes, and showed good timeliness and a predictive value positive (PVP) of 86.2% (personal communication, Yi-Chun Lo, TCDC). As part of quality improvement activities, regular evaluation of the system is needed to ensure that the stated objectives are being achieved. We aimed to assess and describe key attributes of dengue fever surveillance through the NDSS to ascertain the extent to which stated objectives are achieved and to make recommendations for improvements.

## Methods

### The NDSS case definitions of dengue fever

Dengue fever has been a notifiable disease in Taiwan since June 1988. A suspected case is defined as fever (>38°C) with at least one of the following symptoms: retro-orbital pain, myalgia, arthralgia, rash, leukopenia, and haemorrhagic manifestations. A confirmed case is defined as positive DENV identification by virus isolation, nucleic acid testing, non-structural protein-1 (NS-1) antigen testing, or seroconversion [9].

### Operation and resources used

The Communicable Diseases Act in Taiwan makes it a statutory requirement that suspected or confirmed cases of dengue fever be reported via the NDSS to the relevant local health department and TCDC by the diagnosing clinician or laboratory, within 24 hours. The local public health department should initiate patient interviews within 24 hours of receipt of the report to support case finding, contact tracing, and environmental control measures if deemed necessary.

In addition to notifications from physicians, the NDSS also captures data on cases detected through contact investigation, self-reporting, and active surveillance at quarantine stations. Public health professionals actively investigate persons in close contact with a laboratory-confirmed case for symptoms consistent with dengue fever and submit blood samples to TCDC for confirmatory testing. A self-reporting system allows patients with symptoms consistent with dengue fever to present to the local health department for DENV confirmatory testing with a financial incentive of 2,500 new Taiwanese dollars (approximately USD $80) if test results are positive.

Since 2006, a parallel active surveillance system has been in operation to detect febrile inbound passengers by using remote-sensing infrared thermography at quarantine stations of all international airports and harbors. Passengers presenting with fever upon entry from an endemic country are tested for dengue on the spot using an NS-1 antigen test which is sent to TCDC for laboratory confirmation. Laboratory-confirmed cases are automatically reported into the NDSS from the laboratory system feed.

**Fig 1:**
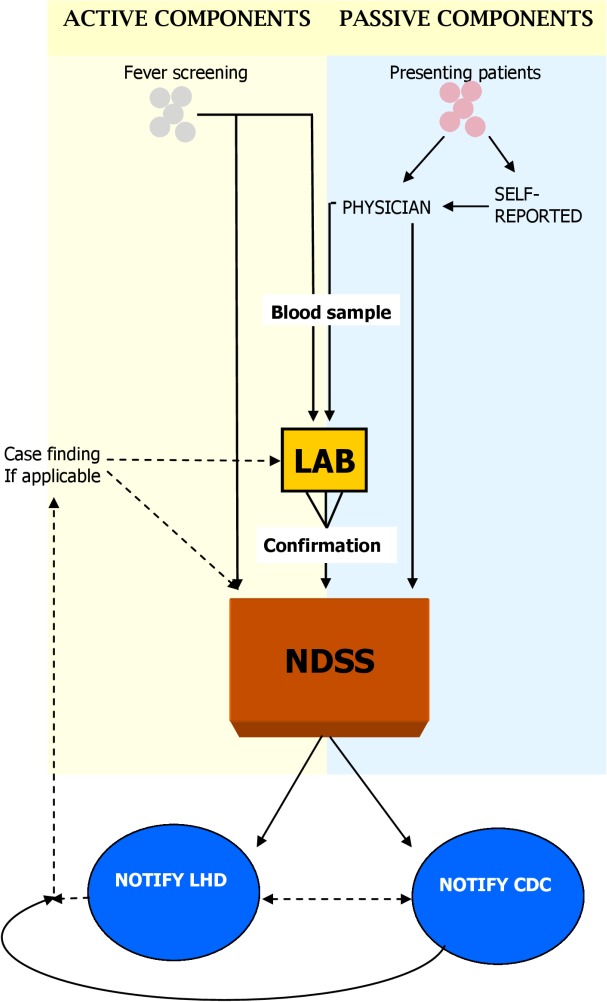
Flow chart of data flows within the National Disease Surveillance System (NDSS) for dengue fever in Taiwan.

Fig. 1 shows the data flow between components of the NDSS. The surveillance data are used to produce a suite of routine and ad-hoc outputs including annual reports, web-based tables and maps, and the Taiwan Epidemiology Bulletin which is disseminated to a range of stakeholders.

The NDSS is operated and maintained by two full-time information specialists, with a back-up computer system located at a separate site. The annual maintenance cost including staff, hardware, and software is approximately two million New Taiwan Dollars and is funded by TCDC. As the NDSS is a universal platform for all notifiable diseases, we cannot determine the proportion of cost attributed to dengue alone.

### Evaluation design

The evaluation design followed the updated guidelines for Evaluating Public Health Surveillance Systems published by the United States Centers for Disease Control and Prevention [10]. Using the stated objectives of the dengue fever surveillance system, appropriate attributes were selected based on the relevance to dengue fever surveillance and the ability to access corroborating information from other systems (e.g. laboratory data). The selected attributes for the evaluation were simplicity, acceptability, data completeness, quality and validity, timeliness, and PVP. Sensitivity, specificity and representativeness were not assessed as time and resource constraints precluded the possibility of obtaining supplementary data from another system and they have been examined in previous evaluations.

### Data collection

Data on all confirmed dengue fever cases reported through the NDSS between 1 January 2010 and 31 December 2012 were anonymized and extracted by TCDC staff. These were translated into English and collated onto a password protected Microsoft Excel database.

### Reporting activity

Reporting activity was explored to describe the epidemiology of cases in Taiwan from 2009–2011. Annual crude and age-specific incidence rates were calculated using age specific census mid-year estimates for the years 2009 to 2011 [11]. Categorical variables were summarised as counts and proportions and continuous variables presented using appropriate measures of central tendency and variation. Exact score mid-p values were ascertained from chi square test for categorical variables and chi square test for trend for continuous data, with a p value of ≤ 0.05 considered statistically significant. All statistical analyses were performed in Stata 12.1 (StataCorp. College Station, USA).

### Assessment of simplicity and acceptability

A 17 item semi-structured questionnaire was used to assess respondents’ knowledge, understanding and views on the current case definitions and objectives of NDSS as well as their views on the relevance, acceptability and ease of use of the system. The questionnaire was administered via face-to-face interviews with internal stakeholders considered key to the operation of the surveillance system. Interviewees were selected based on convenience (availability and willingness to participate) and expertise (minimum of three years’ experience) and interviewed by a TCDC staff member in Mandarin Chinese with the responses recorded in the questionnaire in English.

Simplicity and acceptability were assessed using questions concerning compliance, ease of use, and number of steps in the system alongside users’ opinions on the appropriateness of variables collected and the current case definition. The responses from the interviewees were analysed and summarized in the following way: (1) the number and percentage of respondents providing a ‘Yes’ or ‘No’ response to questions with dichotomous responses; (2) the number and percentage of respondents providing a particular response to questions with scaled (nominal) options; and (3) questions with free text responses in a narrative style without any thematic coding. Completed questionnaires were transcribed into SelectSurvey (SelectSurvey.Net; 2012) and exported to Microsoft Excel for analysis.

### Assessment of data completeness, quality and validity

Completeness was assessed by determining the percentage of case records with recorded data on each variable. Data quality was evaluated by assessing the completeness, validity, and chronological consistency of variables included in the World Health Organization (WHO) recommended minimum data set for dengue fever surveillance [12]. These variables consisted of case classification, unique identifier, patient name, age, sex, geographic information, date of onset, hospitalization, outcome, and travel history during the past two weeks.

Validity was assessed by determining the percentage of case records with valid recorded data on each variable. The validity of travel categorization was assessed by ascertaining the number of cases recorded as travel-related on their patient record. Travel dates were then checked and cases categorized as travel-related using the system definition of “travel outside Taiwan within 3−14 days before illness onset”. Any patient that reported travel outside this window was deemed non-travel related. This was checked against what was recorded in the travel-related field. Chronological consistency was assessed by determining the percentage of case records in which there was consistency between reported date of onset and date of diagnosis.

Guidelines were scare on what represents a good or acceptable level of data completeness or validity among individual fields in surveillance systems. Evaluation is often based on perceived importance of the field in question [13,14]. Using a threshold value of 90% as a satisfactory level of completeness/validity, we reported results as “satisfactory” or “unsatisfactory” if ≥ 90% or < 90% respectively of case records met the threshold value [15].

### Assessment of timeliness and predictive value positive (PVP)

Timeliness was assessed by calculating two metrics; time that elapsed between onset of symptoms to diagnosis and from diagnosis to report. These were summarised as the number of days between these two time points with an appropriate measures of central tendency and variation. The percentage of cases not reported to NDSS within the recommended 24 hours was also calculated. PVP was assessed by calculating the percentage of all tested cases that were laboratory confirmed.

### Ethics statement

Data reported to the NDSS are used for public health surveillance purposes. TCDC determined this study as non-research activity and therefore exempt from the review by the Institutional Review Board at the time it was undertaken (2012−2013). All patient data were anonymized and verbal informed consent was obtained from all interviewees.

## Results

### Reporting activity

**Fig 2:**
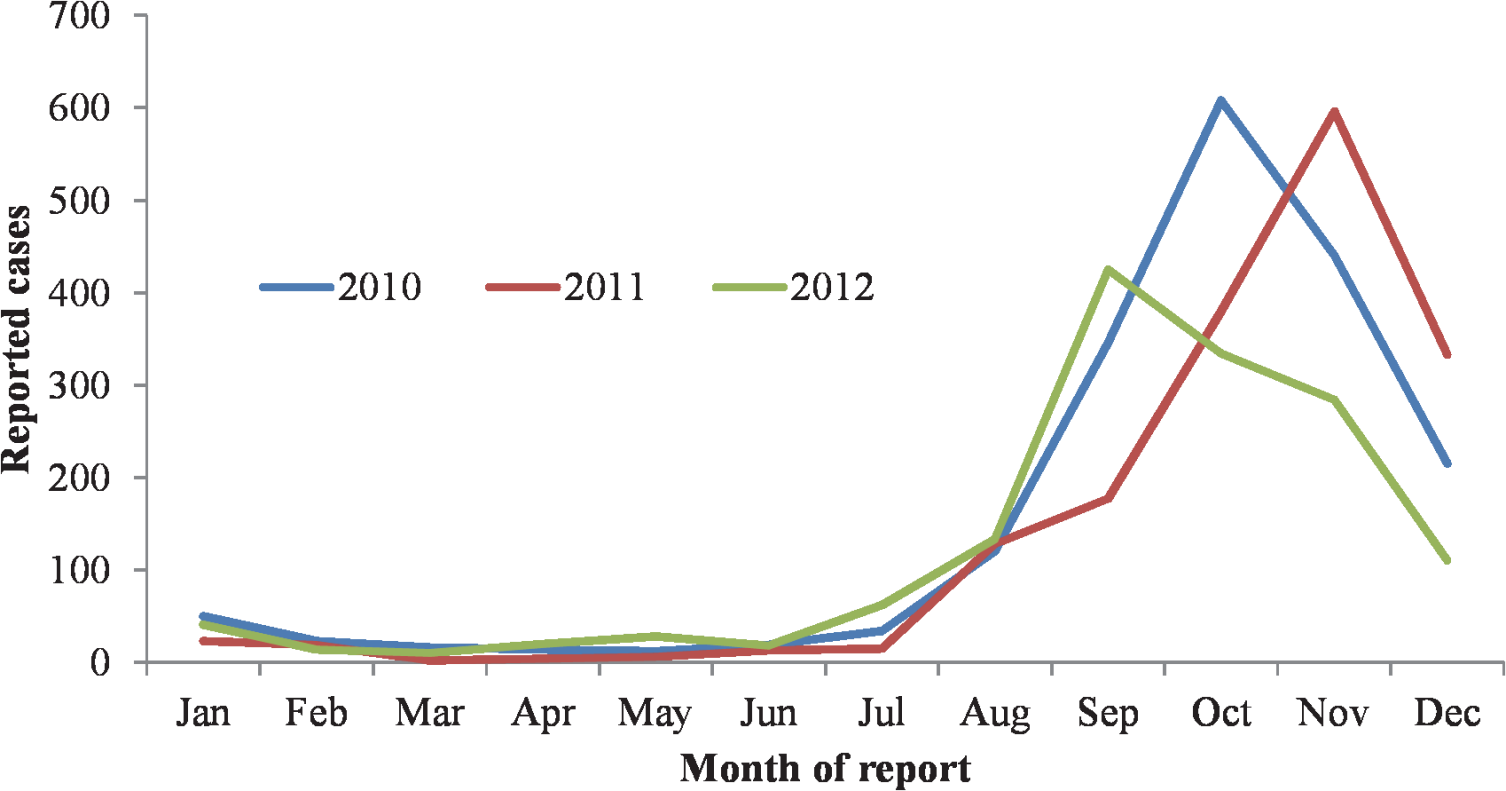
Number of confirmed cases of dengue fever reported to the National Disease Surveillance System by month of report, Taiwan, 2010–2012.

**Fig 3:**
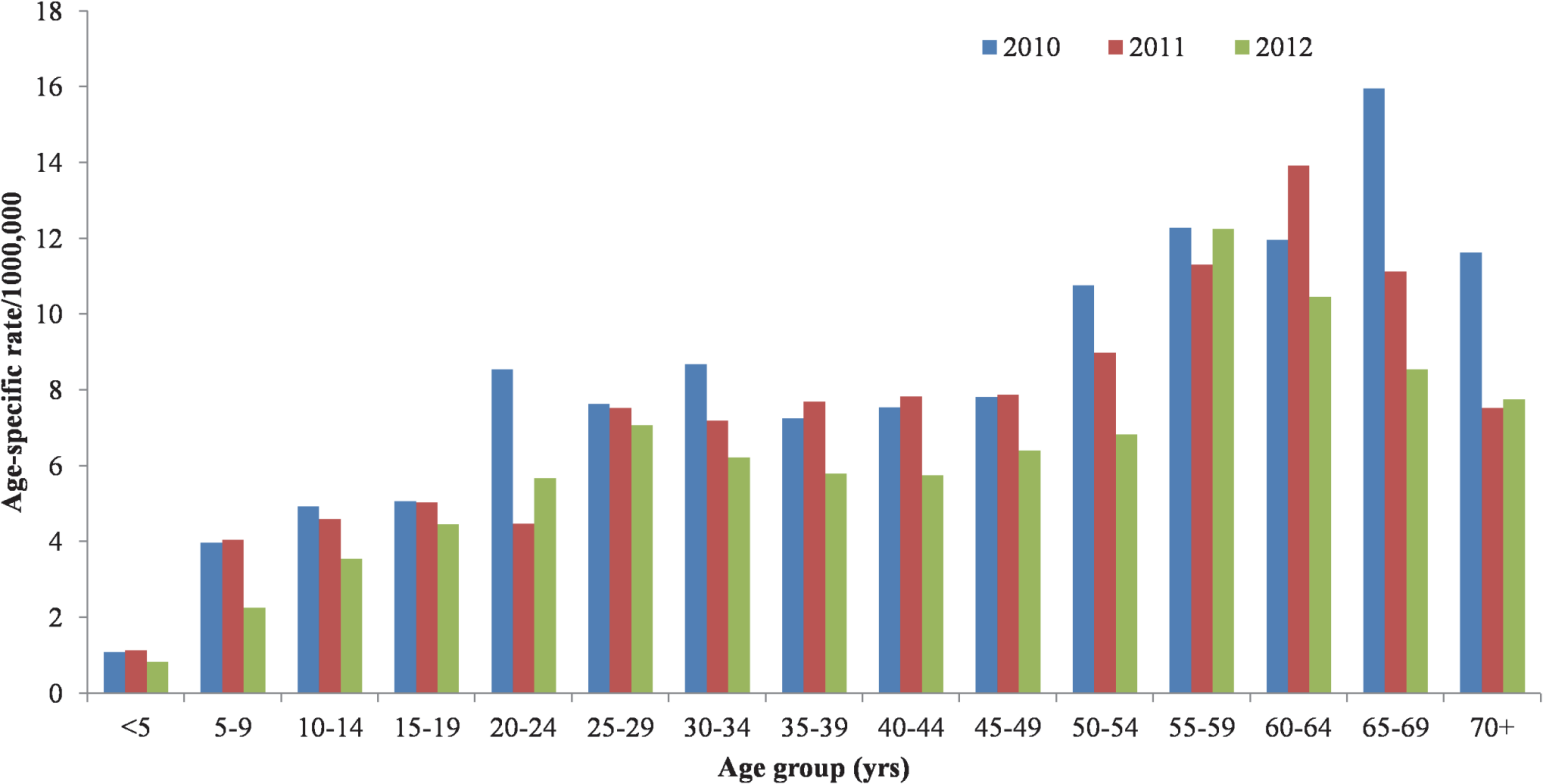
Age-specific dengue fever incidence rates, Taiwan, 2010–2012.

During 2010−2012, 11,718 suspected dengue fever cases were reported to the NDSS; 5,072 were subsequently laboratory confirmed, with annual counts of confirmed cases decreasing over the period (1,897 in 2010, 1,696 in 2011 and 1,479 in 2012). The crude incidence decreased from 8.2/100,000 in 2010 to 6.4/100,000 in 2012. The majority (n = 4,740, 93%) of confirmed cases occurred during July to December (Fig. 2). Of the 5,072 laboratory-confirmed cases, the median age was 45 years (range <1 to 98 years) and 2,530 (49%) were male (Fig. 3).

**Fig 4:**
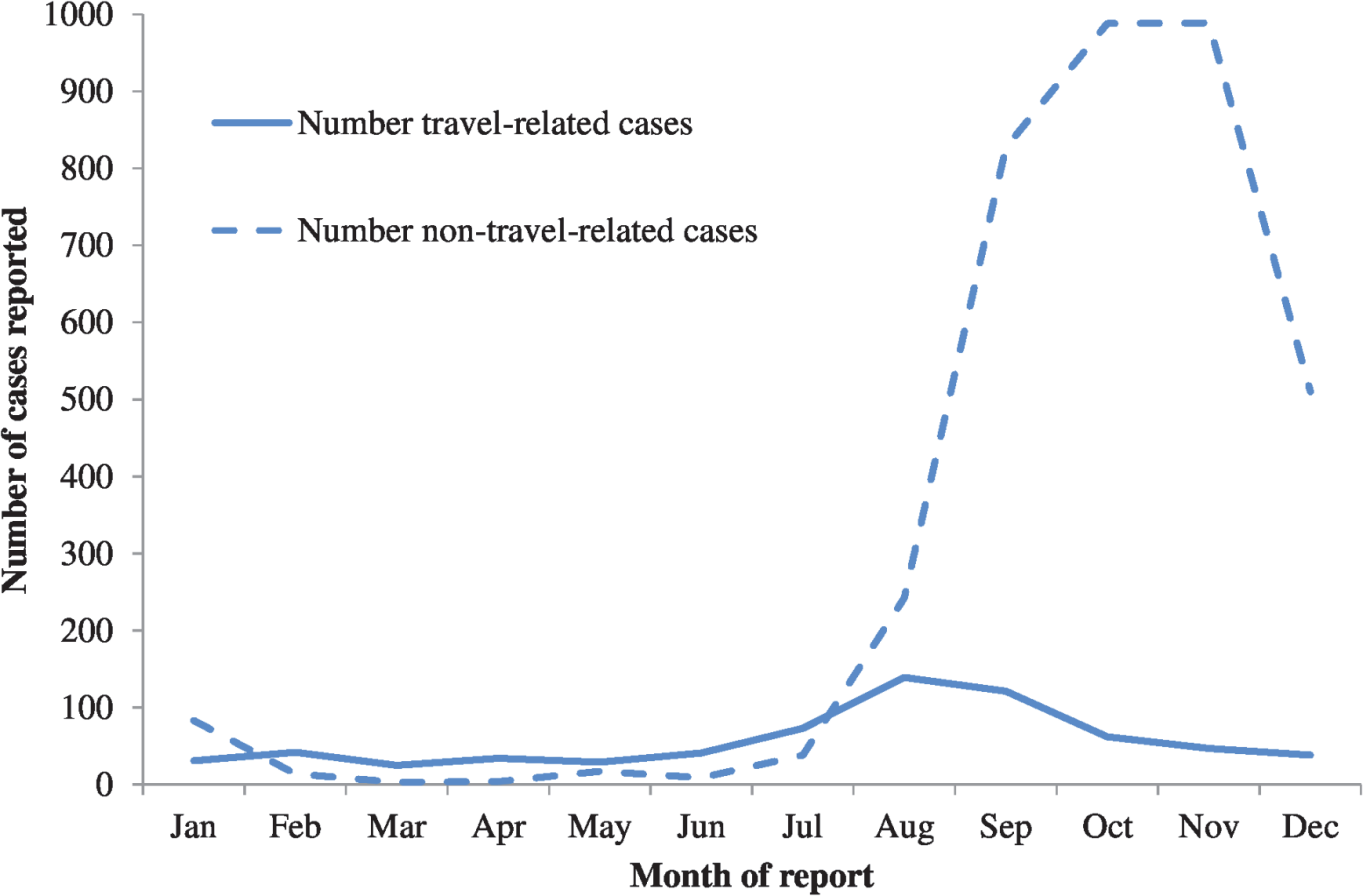
Number of confirmed cases of dengue fever reported to the National Disease Surveillance System by month of report and travel history, Taiwan, 2010–2012.

**Fig 5:**
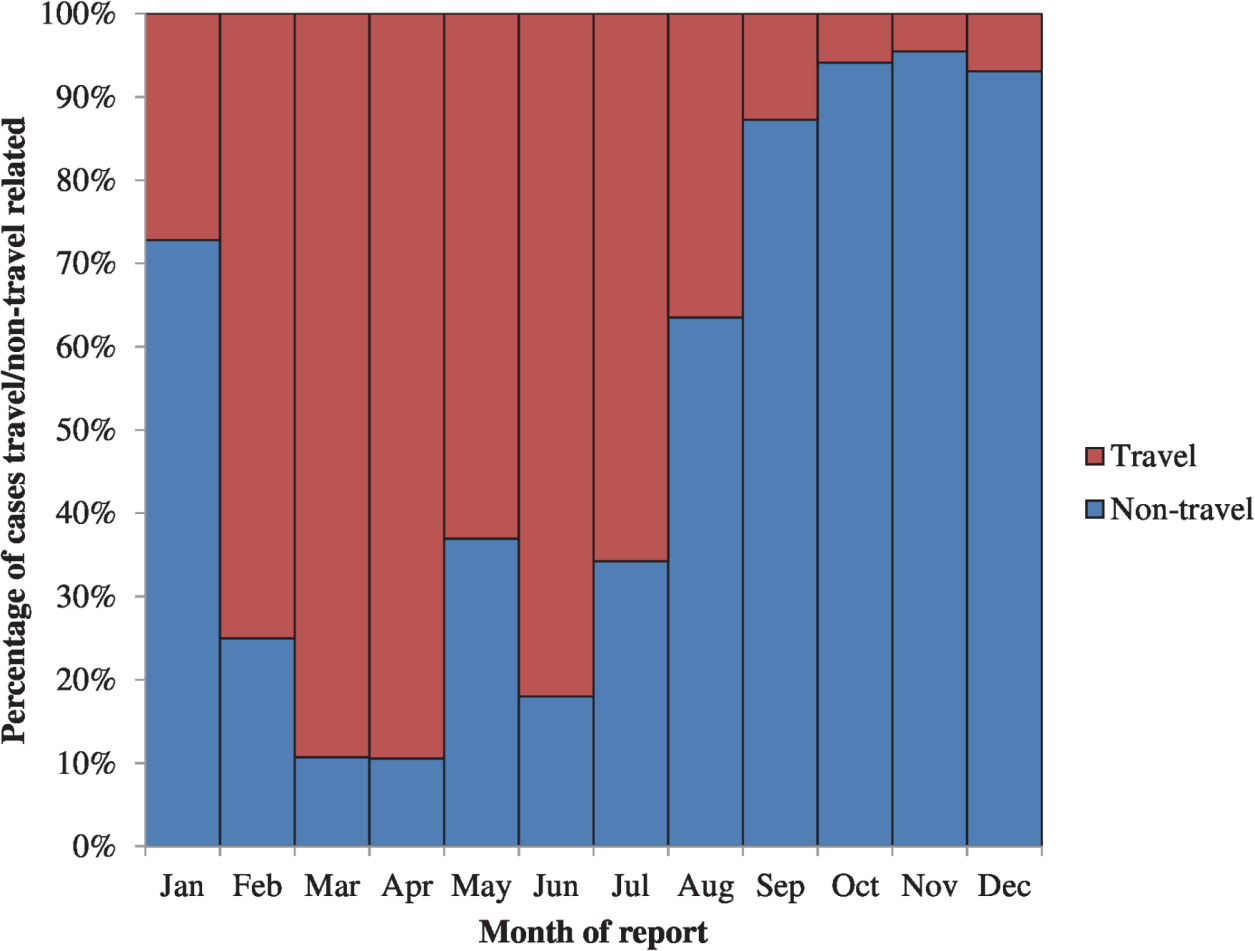
Percentage of confirmed dengue fever cases with/without travel history reported to the National Disease Surveillance System by month of report, Taiwan, 2010–2012.

During the period, 720 (14%) of the 5,072 laboratory-confirmed cases were reported as travel-related and the proportion of travel-related cases did not change significantly annually (p = 0.6). Of the 720 travel-related cases, 111 (15%) reported symptom onset more than 14 days after travel, which did not technically fit the definition of travel-related. Of travel-related cases, 57% were reported during July to October whereas the majority (91%) of non-travel related cases were reported during September to December (Fig. 4 and Fig. 5).

### Simplicity and acceptability

Four individuals participated in the face-to-face interviews; two each from the north and south of the country (three TCDC staff and a member of the local health department). Their job roles were a public health nurse, an infectious disease doctor, an environmental prevention and control officer, and an information technology staff member.

All respondents agreed that the current system was effective or very effective in meeting the objectives of dengue fever surveillance and felt that the variables collected were relevant to dengue fever specifically. All respondents agreed that the dengue surveillance system was clear and easy to use; useful, quick, and that the skills required to use the system existed in their teams. A mixture of uses of the system were reported including case reporting (75%), case monitoring (75%), data analysis (50%), and tracking outbreaks (50%). In addition, two respondents also reported using the system for advice on case management and to identify areas to target for vector surveillance.

Free text comments from respondents revealed that some users felt the case definition was broad and in general, cases were more likely to be diagnosed based on clinicians’ judgment and experience. However, it was accepted that the severity of disease requires a sensitive case definition in order to capture cases.

### Data completeness, quality, and validity

Table 1 summarized completeness of variables reported on confirmed dengue fever cases in the NDSS. The variable “hospitalized” was the only variable with an unsatisfactory level of completeness with 78.5% of records completed. All cases had both an onset date and diagnosis date recorded with no inappropriate values reported (i.e. wrong format or nonsensical values). All recorded onset dates were chronologically consistent with the diagnosis date. All cases had a unique identifier reported. Overall, 99.9% (4810/4817) of cases with Taiwanese residency had a correctly structured unique identifier.

**Table 1:** Percentage completeness of variables in dataset of confirmed dengue fever cases reported to the National Disease Surveillance System, Taiwan, 2010–2012.

Variable Name	Total cases	Number of missing entries	% completeness
ID Number	5,072	0	100
Nationality	5,072	0	100
Travel history	5,072	0	100
Country of travel	5,072	4	99.9
Travel dates	5,072	0	100
Date of birth	5,072	0	100
Age	5,072	0	100
Sex	5,072	0	100
Hospitalized	5,072	1,093	78.5
Onset date	5,072	0	100
Diagnosis date	5,072	0	100
Report date	5,072	0	100
Receipt date (LHD)	5,072	0	100
Receipt date (TCDC)	5,072	0	100

ID, identification; TCDC, Taiwan Centers for Disease Control; LHD, local health department

### Timeliness

The median length of time from symptom onset to presentation for clinical assessment/diagnosis was four days (range 0–49), and 4,556 (90%) of cases were diagnosed within seven days of symptom onset. The elapse between the date of diagnosis to the report date was 0−17 days, with 5,009 (98.8%) reported within the recommended 24 hours.

### PVP

Over the period, 11,796 suspected dengue fever cases were reported to the NDSS with 5,072 subsequently laboratory confirmed. This represents a calculated PVP of 43.0% (Table 2).

**Table 2:** Estimates of predictive value positive of the National Disease Surveillance System for dengue fever by year—Taiwan, 2010–2012.

Year reported	Total cases reported	Total cases tested	Total true cases (Laboratory confirmed)	PVP
2010	4,252	4,251	1,897	44.6%
2011	3,920	3,920	1,696	43.3%
2012	3,624	3,624	1,479	40.8%
**2010–2012**	**11,796**	**11,795**	**5,072**	**43.0%**

PVP, predictive value positive

## Discussion

Our evaluation of dengue fever surveillance in Taiwan shows that the system is simple, acceptable to users and achieves its stated objectives. The high level of completeness of key data fields is encouraging and demonstrates the high quality of the surveillance system but is also an indirect measure of the engagement of stakeholders particularly those who report to the system. The only variable with low reporting was hospitalization information, but this is not a mandatory field and data entry still proceeds even if this is not recorded. Improving the completeness of this variable would be welcomed as it may allow more accurate estimates of severity of illness.

The simplicity of this system and its processes engenders compliance which can facilitate the delivery of effective public health responses and improve outcomes at the individual and population levels [15]. In 2009, changes were made to the NDSS to optimize the surveillance objectives which increased co-operation with other public health services such as laboratory reporting and entomological colleagues in order improve detection of early transmission; and a change in case definition to increase case specificity. These changes have been supported by users of the system and are reflected in the evaluation findings that show stakeholders are engaged and compliant. Engagement can be strengthened by incorporating other forms of surveillance and promote working towards a more integrated response [16–17]. We could not assess the outbreak detection capability of the system but we recommend that future evaluations include this to support the development of statistical algorithms to detect space-time clusters using all available linked data. The sensitivity of such as system may be improved by combining outbreak detection with virologic surveillance [18].

WHO recommend that effective prevention and control of epidemic dengue requires an integrated laboratory based surveillance system that can provide early warning of epidemic transmission [19]. Systems with standardised procedures for laboratory confirmation may improve the ability to distinguish between true cases and other causes of febrile illness.

Our finding that under half (43.0%) of reported cases tested had laboratory confirmed dengue fever represents a considerable decrease from the PVP of 86.2% observed during 2004–2008 (personal communication, Yi-Chun Lo, TCDC). This may be due to the change in the confirmed case definition introduced in 2009 and the low threshold for reporting suspected cases by clinicians. Before 2009, an individual with dengue-like symptoms who was epidemiologically linked to a confirmed case was classified as a confirmed case without the need for further laboratory confirmation. This may have led to an overestimation of the numerator and an artificially inflated estimate of PVP prior to 2009. In addition, PVP may fluctuate during periods of high incidence and the evaluation of other dengue systems have reported that even with increasing PVP there is still a fairly high level of false-positive returns, probably due to sensitive case definitions and a broad spectrum of illness, especially during outbreaks [6].

The calculation and assessment of PVP is unlikely to be robust without incorporating estimates of system sensitivity, which we were unable to undertake in this study due to lack of access to a ‘gold-standard’ data source and so an accurate assessment of this is a key recommendation for further work.

We found that a small number of travel cases were misclassified. To minimize this, we recommend the use of an algorithm to more accurately classify travel cases in order to improve accuracy and ensure adherence to WHO recommended standards for dengue fever [20]. Accurate estimates of imported and autochthonous cases are needed to allow comparisons of the burden of dengue in different geographical locations and time periods and support public health authorities to make informed decisions on resource allocations [21].

There is a possibility of selection bias arising from the convenience sample of stakeholders that were interviewed and this may preclude any generalisation to other key stakeholders. In addition the sample was small and not necessarily representative of the views of the majority of stakeholders. Nonetheless, the views expressed by the respondents are still useful and provided some insight into key aspects of the operation of the system.

Our finding of a predominance of adult cases is consistent with findings from other parts of the region [22–25]. Age is known to be an important predictor of risk and outcome of dengue infection as adults have been found to be at greater risk of hospitalization and death [26]. A number of factors may underpin this observed age distribution including the demographic of travel-related cases and milder symptoms in children.

Our study has identified a number of key recommendations including a further assessment of sensitivity and PVP, ability of all parts of the system to accurately capture and track outbreaks and efforts to improve data quality and completeness to provide a clearer picture of severity and actual burden of imported cases. These are consistent with the recommendations from a facilitated discussion involving members of the Dengue Prevention Board in the Asia-Pacific and America that include: collection of a minimum data set; additional studies to verify the sensitivity of the system; data sharing with laboratories; and goals for national surveillance systems to include early detection and prediction of dengue outbreaks [15].

This study had some limitations due to lack of access to data which meant that we were unable to undertake an assessment of estimates of system sensitivity. Also, the evaluation of simplicity and acceptability doesn’t cover cases’ perception and use of the reporting systems, only public health staff. Staff members were interviewed based on convenience due to availability, English language ability, and willingness. This could bias the sample and led to small numbers available for interview, leading to a less representative sample than is usually required. Issues with providing data to non-Taiwan CDC staff meant that the available dataset was only available for anonymous variables, and only case information; we were unable to investigate any linked data such as vector information or serological results.

In conclusion, our study shows that comprehensive dengue fever surveillance is necessary for establishing a clear picture of the local burden and distribution of disease in populations, in order to effectively support public health response. The NDSS is a robust and valid integrated system which supports these efforts and assists with the reporting of a clear picture of the epidemiology of dengue fever in Taiwan. Integrated surveillance and outbreak preparedness remain central enabling factors that highly contribute to effective implementation of the global strategy to reduce the burden of dengue infection worldwide and further efforts to improve these should be on-going [19].
